# Tubercular Ulceration of the Tongue

**Published:** 1887-05

**Authors:** 


Tubercular Ulceration of the Tongue: Treatment by Eucalyptol and Lactic Acid.—Dr. Poncet, in the *Lyon Medical*, January, 1887, reports a case of a patient presenting tuberculosis of the right apex, similar ulcerations of the tongue, and digital ulcerations of the same nature. Tubercle bacilli were found in these latter. The patient has been under a course of treatment by eucalyptol and lactic acid ( eighty per cent). The ulcerations of the fingers were dressed with lint dipped in eucalyptol. Twelve to fifteen days afterwards the wounds were nearly completely cicatrized; but this was only in appearance, since the skin was infiltrated with fungosities below the epidermic secretion. Dr. Poncet thinks that eucalyptol acts as a desiccator of the secretions, but there is no proof that it is a microbe destroyer. As to lactic acid used for the ulcerating of the tongue, it produced much pain, lasting for an hour after the application, but no appreciable amelioration of the condition.—*Journal of Laryngology and Rhinology.*

